# Purification of thonningianins A and B and four further derivatives from *Thonningia sanguinea* by one‐ and two‐dimensional centrifugal partition chromatography

**DOI:** 10.1002/jssc.201900811

**Published:** 2019-11-11

**Authors:** Luca Pompermaier, Stefan Schwaiger, Monizi Mawunu, Thea Lautenschlaeger, Hermann Stuppner, Karine Faure

**Affiliations:** ^1^ Institute of Pharmacy/Pharmacognosy Center for Molecular Biosciences Innsbruck University of Innsbruck Innsbruck Austria; ^2^ Kimpa Vita University Uíge Angola; ^3^ Department of Biology Institute of Botany, Faculty of Science Technische Universität Dresden Dresden Germany; ^4^ Institut des Sciences Analytiques Université de Lyon CNRS Université Claude Bernard Lyon 1 Villeurbanne France

**Keywords:** centrifugal partition chromatography, dihydrochalcone glycosides, ellagitannins, preparative chromatography, thonningianin A

## Abstract

*Thonningia sanguinea* is a parasitic herb widely used in traditional African medicine. Dihydrochalcone glucosides (unsubstituted, substituted with hexahydroxydiphenoyl or galloyl moieties) are the main constituents in the subaerial parts of this plant. In the present study, purification of the six major compounds from a methanol extract of the plant's subaerial parts was achieved by centrifugal partition chromatography. A first dimension centrifugal partition chromatography separation with the solvent system methyl tert‐butyl ether/1,2‐dimethoxyethane/water (1:2:1) in the ascending mode enabled the isolation of the two major bioactive compounds thonningianin A and B from 350 mg of methanol extract within only 16 min with respectable yields (25.7 and 21.1 mg), purities (87.1 and 85%), and recoveries (71.2 and 70.4%). Using a multiple heart‐cutting strategy, the remaining four major dihydrochalcone glucosides of the extract were further separated in a second dimension centrifugal partition chromatography with the solvent system ethyl acetate/1,2‐dimethoxyethane/water (2:1:1) in the descending mode with high purities (88.9–98.8%).

**Article Related Abbreviations**
CPC: centrifugal partition chromatography
DME: 1,2‐dimethoxyethane
MTBE: methyl tert‐butyl ether
thA: thonningianin A
thB: thonningianin B

## 1 INTRODUCTION

*Thonningia sanguinea* is a widely used medicinal plant throughout tropical Africa 1, 2, 3. During an ethnopharmacological survey in the province of Uíge in northern Angola, its various traditional uses have emerged, for example, the use against erectile dysfunction, cough, urinal infections, and as an anthelmintic 4. In a recent study on this plant, we reported the in vitro pharmacological potential of *T. sanguinea* and some of its secondary metabolites as inhibitors of protein tyrosine phosphatase 1B (PTP1B) 5, a cellular receptor representing a potential target for the treatment of diabetes, obesity, and some types of cancer 6. The crude methanol (MeOH) extract of the plant batch investigated in the present study contained six dihydrochalcone glucosides as major compounds, which were identified as thonningianin A (**thA**), thonningianin B (**thB**), 3‐hydroxyphloridzin (**1**), 2′‐*O*‐(6‐*O*‐galloyl‐*β*‐d‐glucopyranosyl)‐3‐hydroxyphloretin (**2**), 2′‐*O*‐(4,6‐*O*‐*S*_a_‐hexahydroxydiphenoyl‐*β*‐d‐glucopyranosyl)‐3‐hydroxyphloretin (**3**), and 2′‐*O*‐(3‐galloyl‐4,6‐*O*‐*S*_a_‐hexahydroxydiphenoyl‐*β*‐d‐glucopyranosyl)‐3‐hydroxyphloretin (**4**) 5, 7. Except for (**1**), all mentioned compounds comprise galloyl groups or a hexahydroxydiphenoyl group at their glucose moiety and can thus be defined as hydrolysable tannins. For a more detailed investigation of the faith of the compounds after oral ingestion and the corresponding bioavailability, the isolation of the main compounds within a short time and with high purity is of special interest.

Countercurrent chromatography (CCC) is an overarching term for all forms of liquid–liquid chromatography that use a biphasic immiscible liquid system without any solid support for separation. In CCC, the liquid stationary phase is retained in the device by centrifugal forces, while the liquid mobile phase is pumped through. CCC qualifies mainly as preparative technique for two reasons: on one hand, the separation power is less affected by the injection of large sample amounts than that of a solid‐phase LC column (overloading effects). On the other hand, the performances (efficiency and stationary phase retention) are improved during the scale‐up process 8. Further benefits of CCC are the possibility to recover all components injected into the system, thanks to extrusion and the absence of irreversible adsorption or sample contamination through interaction with a solid stationary phase. Therefore, it is particularly suited for the separation of complex samples such as plant extracts and plays an important role in natural products chemistry 9, 10, 11.

In the past, CCC has been used for the separation and purification of common hydrolysable tannins 12, 13, 14 as well as for the isolation of dihydrochalcone glucosides from Sweet Tea (*Lithocarpus polystachyus*) 15. These separations were conducted over 6 h for up to three targets. The lead compounds of the extract investigated in this study represent hybrids of these two compound classes, which have not been subjected to separation with CCC related methods until now. The aim of this study was the development of a multiple heart‐cutting 2‐D CCC method to provide the purification of the six major constituents of the MeOH extract of *T. sanguinea* subaerial parts in a timely manner, with the focus on the fast isolation of the major two biologically active constituents, **thA** and **thB**. For that purpose, a first CCC dimension (^1^D) was carried out at large scale in a normal phase mode and a second CCC dimension (^2^D) was performed to separate selected fractions in a reverse phase mode. To ensure a fast process, an instrument with a hydrostatic CCC design, a centrifugal partition chromatograph (CPC), was used. CPC devices generally can be operated with almost any two‐phase system, are resistant to stationary phase loss, and allow high flow rates and hence short separation times 16.

## 2 MATERIALS AND METHODS

### 2.1 Chemicals and instrumentation

All solvents used for extract preparation, HPLC analysis, and CPC separations were of analytical grade and were purchased from Sigma‐Aldrich (Isle d'Abeau, France). 1,2‐Dimethoxyethane (unstabilized) was purchased from TCI (Zwijndrecht, Belgium) and methyl tert‐butyl ether from Acros Organics (Fischer Scientific, Illkirch, France). Standards of the target compounds used for the identification of our isolates were obtained by conventional chromatographic methods during our previous study on *Thonningia sanguinea* 5. The laboratory scale CPC instrument used in this study was the FCPC‐A from Kromaton Rousselet‐Robatel (Annonay, France) with interchangeable rotors. The rotors had an exact volume of 253 mL for ^1^D large‐scale and 36 mL for ^2^D small‐scale separation experiments. The CPC apparatus was equipped with a PuriFlash1000 (Interchim, Montluçon, France) providing the quaternary pump, UV detector set at 280 nm, an automatic sample injection valve fitted with a sample loop (1.08 mL for small scale, 18.9 mL for large‐scale experiments), and a fraction collector. The HPLC system used for the analysis of all fractions was an Alliance 2690 from Waters (Saint‐Quentin‐en‐Yvelines, France) using a binary pump, an autosampler, and a DAD detector Waters 996. Waters EmPower software was used for data acquisition.

### 2.2 Plant material and extract preparation

**Figure 1:**
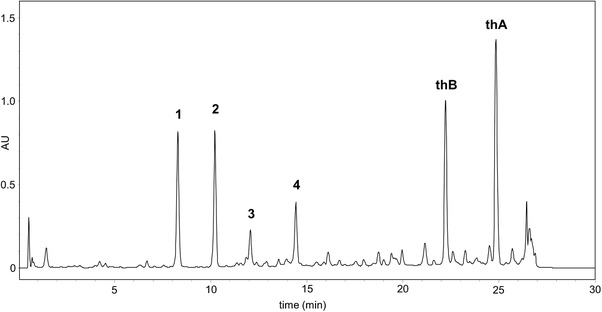
HPLC‐UV chromatogram (280 nm) of the defatted MeOH extract (5 mg/mL MeOH) of *Thonningia sanguinea* subaerial parts. For compound structures, see Figures 2 and 3. The remaining HPLC parameters are summarized in the materials and methods section

The underground parts of *Thonningia sanguinea* Vahl (Balanophoraceae) were collected in a forest in November 2016 during a field trip in the northern Angolan province of Uíge (S 7°04′32.5″, E 14°38′14.3″, 541 m. a.s.l.). The collection and export permits were issued by the Ministry of Environment of Angola and the Province Government of Uíge. The collected plant material was identified in Dresden, Germany. An authenticated plant voucher specimen is deposited at the herbarium of the Technische Universität Dresden (voucher no. HD 043263). The collected plant material was cleaned, immediately dried in a drying cabinet at 40°C (HTD 100 Bench Top, LinTek), and subsequently boxed using a vacuum machine. For extract preparation, the dried plant material was milled to a fine powder using an electric coffee grinder and 5 g thereof weighed into an Erlenmeyer flask. To remove surface‐active compounds that could disturb the biphasic liquid system in CPC (e.g. fatty acids, triglycerides), the material was first defatted by sonication with petroleum ether (5 min with 30 mL, 4 times). The residue was dried and extracted with 30 mL MeOH using sonication for 5 min. The procedure was repeated six times with fresh MeOH to ensure exhaustive extraction, the extracts were pooled, and the solvent subsequently removed by vacuum rotary evaporation to yield 2.71 g of dry extract. An HPLC‐UV chromatogram of the defatted crude extract is shown in Figure 1.

### 2.3 Selection of the CPC solvent systems

Partition coefficients of the six major compounds and some prominent impurities were determined using the shake‐flask method: 20 mg of the extract was weighed into a small flask and 2 mL of each of the phases from the equilibrated solvent system was subsequently added. The mixture was shaken vigorously for several seconds to allow the complete dissolution and equilibration of the compounds between the two phases. Thereafter, 1 mL of both phases were separately transferred into HPLC vials. While the lower (aqueous) phase was directly analyzed by HPLC, the solvent of the upper phase was removed by evaporation with N_2_, and the residue redissolved in 1 mL MeOH prior to HPLC analysis. The distribution coefficients were finally obtained by calculating the ratio of the peak areas at 280 nm of each target compound in the two phases.

### 2.4 Centrifugal partition chromatography

The solvent systems for the CPC runs were prepared by mixing and equilibrating the respective solvents directly in HPLC glass bottles. Samples for the CPC were dissolved in an equal mixture of upper and lower phase of the respective solvent system and filtered through a 0.45 µm PTFE filter before injection. Prior to each CPC run, the system was filled with two rotor volumes of stationary phase and equilibrated at the selected rotation speed by pumping the mobile phase at the indicated flow rate. Stationary phase retention volume ratios (S_f_) were monitored either by collecting the volume of the displaced stationary phase after equilibration or by considering that unretained compounds, providing a slight increase in UV signal, exhibit an elution volume equal to the mobile phase volume.

The ^1^D large scale CPC was used with the solvent system MTBE/DME/water (1:2:1) at 2000 rpm, ascending mode, 20 mL/min. The sample was 350 mg MeOH extract dissolved in 9 mL upper/lower phase mixture (1+1), which was injected in the sample loop filled with mobile phase. Detection was performed at 280 nm. The eluate was collected every 10 mL up to 19.5 min, then every 15 mL. Each tube was analyzed by HPLC.

**Figure 2:**
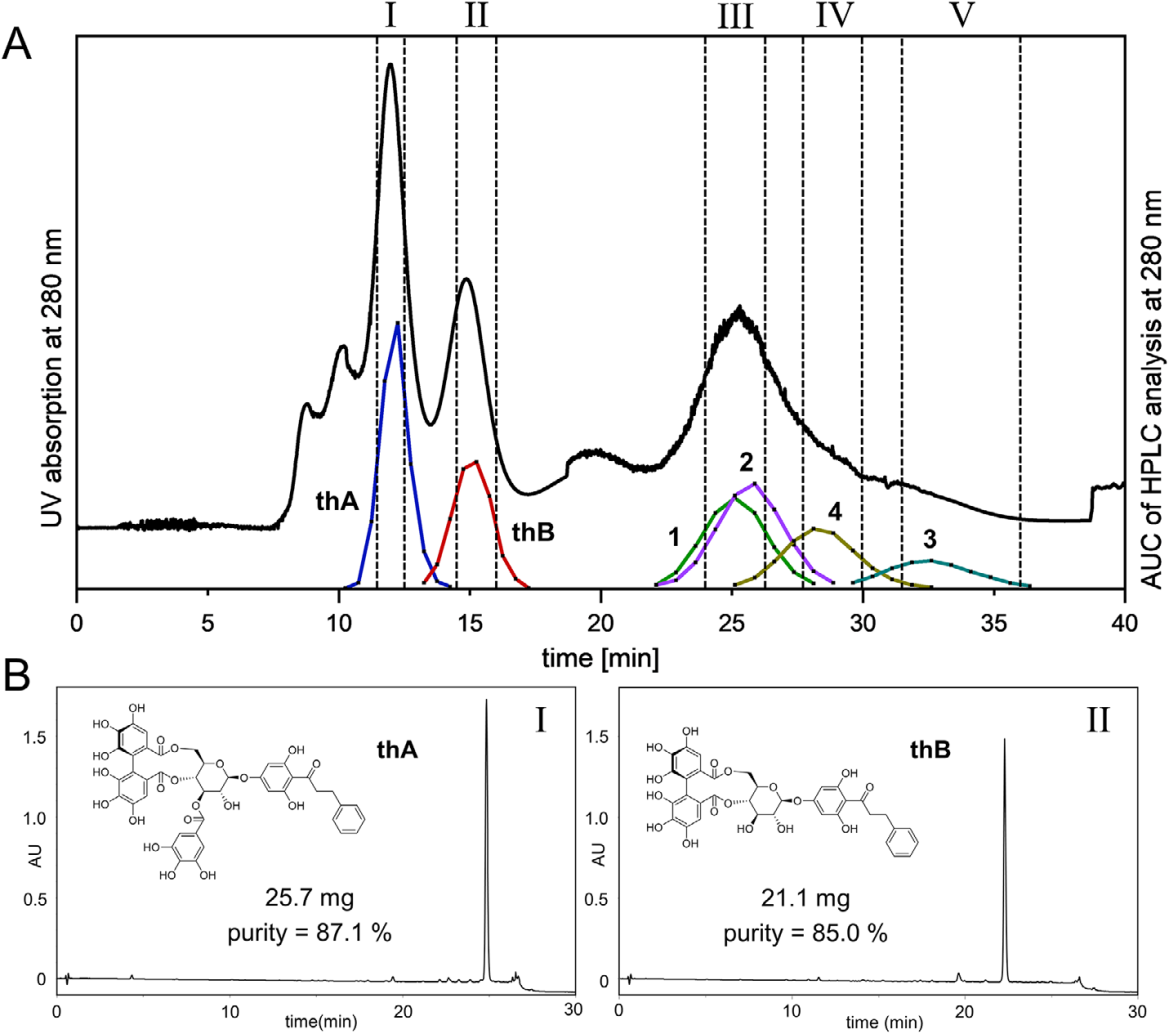
(A) ^1^D CPC separation of the MeOH extract on the 253 mL rotor; sample concentration: 350 mg in 9 mL upper/lower phase mixture; *S*_f_ = 64.4% prior to and 39.9% after injection; 58 bar; black line: CPC‐UV chromatogram; colored lines: peak reconstruction of the target compounds utilizing AUC of the HPLC analysis. Fractions I–V according to the dashed lines. (B) Chromatograms of the HPLC analysis of combined fractions I (thA) and II (thB), at 210 nm; purity according to HPLC‐UV

Three fractions (III, IV, V) were respectively pooled as shown in Figure 2A, evaporated to dryness and sent to the ^2^D CPC with the solvent system ethyl acetate/DME/water (2:1:1). The small rotor was used in descending mode, at 3 mL/min, 2800 rpm. Samples of 16.0 (III), 13.1 (IV), and 13.0 (V) mg of the respective fraction were dissolved in 1.08 mL upper/lower phase mixture (1+1). Fraction collection was performed every 3 mL (1 min).

### 2.5 HPLC analysis

All HPLC assays were performed on an Agilent Eclipse XDB‐C_18_ 3.5 µm, 3 × 100 mm column at 1 mL/min, 20°C, 280 nm. Mobile phase: (A) water + 0.02 % trifluoroacetic acid, (B) ACN. Gradient 10% to 40% B in 25 min.

All six target compounds were identified in the fractions by comparison of their retention times with authenticated standards. For purity estimation of the compounds in the pooled fractions, 5 µL of the sample solutions (1 mg/mL) was analyzed at 210 nm. The purity reflects the percentage of the peak area of the target peak vs. the total integration area throughout the chromatogram. The contents of the target compounds (**1**)–(**4**), **thA**, and **thB** in the used extract were determined using a validated UHPLC method previously published 7. Therefore, three solutions of 15 mg defatted MeOH extract in 25 mL MeOH were prepared and subsequently assayed in three replicates each. The recoveries of the target compounds were estimated based on the following equation:

(1) $$\%\;\mathrm{compound}\;\mathrm{recovery}=\frac{\mathrm{weight}\;\mathrm{of}\;\mathrm{the}\;\mathrm{fraction}\;\mathrm{containing}\;\mathrm{the}\;\mathrm{target}\;\mathrm{compound}\times \%\;\mathrm{purity}}{\mathrm{amount}\;\mathrm{of}\;\mathrm{target}\;\mathrm{compound}\;\mathrm{in}\;\mathrm{the}\;\mathrm{used}\;\mathrm{extract}}$$

For monitoring the tube content obtained from the ^1^D CPC and for peak reconstruction in Figure 2A, 1 mL of the eluate was evaporated to dryness, reconstituted in MeOH, and an aliquot (5 or 10 µL) was analyzed by HPLC. For the ^2^D CPC, 10 µl of the eluate was directly injected for HPLC analysis of each collected tube.

## 3 RESULTS AND DISCUSSION

### 3.1 Solvent system selection for first dimension centrifugal partition chromatography separation

The quite complex HPLC chromatogram of the defatted MeOH extract used in these investigations (Figure 1) displays six major and several minor compounds. The goal of this study was to accomplish the purification of the six major compounds (**1–4**, **thA,** and **thB**) by CPC, with the focus on a fast purification of the two bioactive substances **thA** and **thB**.

The first step of CCC method development consists in the selection of a suitable solvent system, in which target compounds have distribution coefficients (*K*) in an acceptable range that would allow to elute them in one step, i.e. *K* between 0.1 to 8 17, along with separation factors α (α = *K*_2_/*K*_1_) > 1.5 in order to guarantee a successful separation. The partition coefficients of the six target compounds (**1–4**, **thA**, and **thB**) of the defatted MeOH extract in a series of solvent systems are shown in Table 1. The *K* values of the most prominent impurities were also calculated and considered (data not shown). In a previous CCC separation of hydrolysable tannins, a solvent system consisting of *n*‐butanol, *n*‐propanol, and water has been used 12. Applied on the MeOH extract of *T. sanguinea*, this solvent system showed either strong emulsification (ratio 4:1:5) or poor distribution (ratio 2:1:3, see Table 1). In the two‐solvent system water/*n*‐butanol (1:1), the *K* values of the compounds were close to  ∞ (data not shown), indicating that a less polar solvent combination could be more appropriate. In solvent systems of the “Arizona” series (heptane/ethyl acetate/MeOH/water), the compounds showed marginal distribution in the two phases, but with *K* values lying mostly outside of the practicable range. Finally, the most suitable *K* values were obtained with a solvent system containing methyl tert‐butyl ether (MTBE), 1,2‐dimethoxyethane (DME), and water in a ratio of 1:2:1 (No. 10, see Table 1). The settling time of the solvent system when containing the sample was 40 s (40 mg extract/mL) and no emulsification was observed. Moreover, the two priority compounds **thA** and **thB** exhibited *K*_asc_ values below 2 with a large selectivity, indicating that their CPC separation in ascending mode (aqueous stationary phase) should be fast. Therefore, this solvent system was selected for the ^1^D CPC separation.

**Table 1:** Partition coefficients of **1**–**4**, **thA,** and **thB** in selected solvent systems, measured using the shake‐flask method

				Compounds and their respective K values					
No.	Solvent system	Partition coefficient	Composition (v/v)	**1**	**2**	**3**	**4**	**thB**	**thA**
1	*n*‐butanol/*n*‐propanol/water	K_des_	2:1:3	6.9	11.6	7.7	15.9	39.0	110
2	heptane/ethyl acetate/MeOH/water	K_des_	1:3:1:3	0.15	0.21	0.10	0.36	1.92	7.92
3	heptane/ethyl acetate/MeOH/water	K_des_	1:4:1:4	0.28	0.49	0.29	1.35	5.46	32.6
4	heptane/ethyl acetate/MeOH/water	K_des_	1:5:1:5	0.44	0.85	0.58	2.76	10.3	82.3
5	heptane/ethyl acetate/MeOH/water	K_des_	1:5.5:1:5.5	0.50	1.09	0.76	4.32	17.2	∞
6	MTBE/water	K_des_	1:1	0.47	1.32	0.36	6.22	4.55	58.4
7	MTBE/ACN/water	K_des_	4:1:5	0.57	1.41	0.91	7.02	3.6	20.1
8	MTBE/ACN/water	K_des_	2:2:3	1.31	2.59	2.97	11.2	18.9	87.1
9	MTBE/DME/water	K_des_	1:1:1	7.17	7.47	19.8	10.3	1.74	0.68
10	MTBE/DME/water	K_asc_	1:2:1	3.76	4.01	6.44	5.07	1.39	0.78
11	MTBE/DME/*n*‐butanol/water	K_asc_	1:1.75:0.25:1	2.19	2.11	3.08	2.37	0.91	0.53
12	ethyl acetate/DME/water	K_des_	2:1:1	0.96	1.55	1.41	3.25	9.95	31.5
13	ethyl acetate/DME/water	K_des_	1.5:1:1	0.78	1.17	1.07	2.18	6.86	20.0

### 3.2 First dimension centrifugal partition chromatography separation

The ^1^D CPC was performed in ascending mode, meaning that the lower phase of the chosen solvent system (MTBE/DME/water, 1:2:1) acted as stationary phase and the upper phase as mobile phase. A rotation speed of 2000 rpm and a flow rate of 20 mL/min applied on the large CPC rotor afforded a stable stationary phase volume ratio (*S*_f_) of 64% in the column after equilibration with a maximum backpressure of 62 bar. A sample solution close to the solubility limit (38.8 mg/mL) was injected into the 9 mL loop representing an amount of 350 mg. Despite a significant stationary phase, bleed after injection (estimated S_f_ 39.5 %), nearly resolved symmetric peaks (Rs 1.31) could be observed for **thA** and **thB** in the CPC‐UV chromatogram (Figure 2A) in a relatively short elution time (below 18 min). The pooled fraction I afforded 25.7 mg of **thA** in a purity of 87.1% within 12.5 min after injection of the crude extract, whereas 21.1 mg of **thB** (purity = 85%) were obtained from fraction II after 16 min. Corresponding HPLC analyses are shown in Figure 2B. The compound recovery was 71.2% for **thA** and 70.4% for **thB**. Since the obtained purity might be not sufficient for some purposes, additional experiments were performed to increase the purity by Sephadex LH20 column chromatography (see Supporting Information S3–S6) resulting in an increase of the purity to 95.7% (**thA**) and 90.1% (**thB**), respectively.

The injection of a higher sample amount (780 mg) was also investigated but led to a more dramatic loss of stationary phase (*S*_f_ decreased from 64 to 33%) accompanied by a loss of resolution (Supporting Information Figure S1). Nevertheless, **thA** and **thB** could still be obtained with declined purities (79.6 and 81.2%), but with increased yields (51.1 and 52.3 mg) and only slightly decreased recoveries (63.4 and 63.5%), which underlines the robustness of this CPC method.

The remaining four target compounds (**1–4**) eluted as estimated from their *K* values. The selectivity between these targets was estimated below 1.3 during shake‐flasks experiments and hence, as expected, they were not separated from each other by CPC. While a second dimension is then compulsory to separate these compounds, it is of interest to build on the preliminary separation. Hence, a multiple heart‐cutting strategy was chosen by selecting fractions in which the number of targets is limited. Therefore, the eluate of the ^1^D CPC was combined to fractions III (min 24:00–26:15), IV (min 27:45–30:00), and V (min 31:30–36:00) according to the HPLC profile of the single tubes (shown in Figure 2), taking in consideration co‐eluting minor impurities as well. Subsequently, those fractions were purified by a ^2^D CPC exhibiting a different selectivity.

### 3.3 Second dimension centrifugal partition chromatography separations

**Figure 3:**
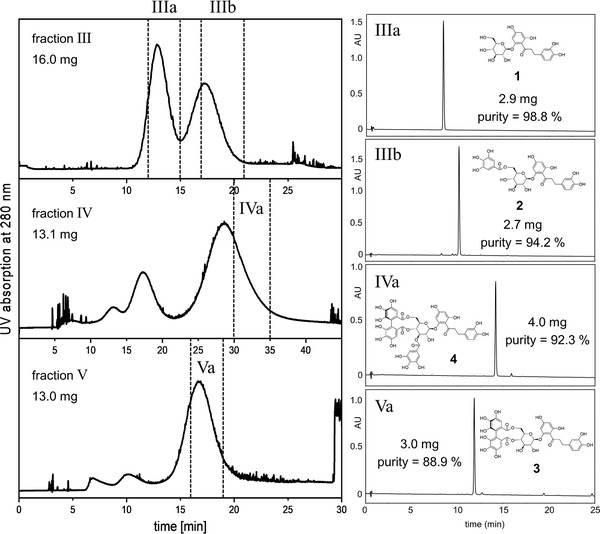
^2^D separation of the fractions III–IV on the 36 mL CPC rotor; left side of the figure: CPC‐UV chromatograms (*S*_f_ = 45.8–47.2%; 49–51 bar). Right side: chromatograms of the HPLC analysis of the pooled fractions IIIa, IIb, IVa, and Va. (210 nm), which were combined according to the dashed lines; purity according to integration of the chromatogram

In order to isolate compounds **1–4** from fractions III‐V of the ^1^D CPC, the implementation of a second CPC method was necessary. The experiments were conducted on a smaller rotor (36 mL), as the amount of the fractions enriched in the target compounds (III–V) was comparably small. The solvent system ethyl acetate/DME/water (2:1:1, No. 12, Table 1) used in descending mode, i.e. the stationary phase is the organic upper phase, featured *K*_des_ values for **1–4** in an acceptable range, with the target compounds eluting according to a reverse‐phase mechanism. Similar to the ^1^D, the presence of the aprotic di‐ether DME turned out to be essential for a satisfying distribution of the compounds between the two phases. The exchange of MTBE with ethyl acetate provided an additional selectivity which can be easily illustrated by an x,y‐plot of the corresponding *K* values of each compound obtained of system No 10 (x‐axis) vs. system No 12 (y‐axis) (Supporting Information Figure S2). Thus, the separation factor α of compounds **1** and **2** was improved up to 1.6, while the selectivity between **2** and **4** reached a value of 2.1. As expected, the separation of the three co‐eluting compounds of the ^1^D CPC was achieved in the ^2^D CPC as shown in Figure 3. Compounds **1** and **2** were separated by ^2^D CPC of fraction III. Compound **4** could be obtained from fraction IV and finally, compound **3** was separated from impurities by ^2^D CPC of fraction V. The selection of a short collection interval, taking into consideration minor impurities in the HPLC profile of the monitored tubes, enabled the production of highly pure compounds with the disadvantage of a rather low recovery (11.4, 12.6, 40.4, and 33.7% for **1**, **2**, **3**, and **4**, respectively). For an increase of recovery, the collection window might be enlarged, however with the drawback of lower purity.

## 4 CONCLUDING REMARKS

In the presented study, six major dihydrochalcone glucoside derivatives could be obtained with high purity (≥85%) from a complex MeOH extract of the subaerial parts of *T. sanguinea* by the sole use of CPC in a timely manner. The two bioactive substances **thA** and **thB** were purified within just 16 min with the ^1^D CPC solvent system in the ascending elution mode with good yields (25.7 and 21.1 mg) and respectable purities (87.1 and 85%) and recoveries (71.2 and 70.4%) on a 253 mL CPC rotor.

In addition, a multiple heart‐cutting strategy was performed on selected fractions with a ^2^D CPC introducing additional selectivity on compounds **1**–**4**. The overall purification of the six major compounds of *T. sanguinea* subaerial parts could hence be performed in few hours.

This study underlines the versatility of centrifugal partition chromatography in natural product purification and once again emphasizes this technique as useful alternative to separation techniques relying on solid stationary phases.

## CONFLICT OF INTEREST

The authors have declare no conflict of interest.

## Supporting information

Supporting Information
