# Supplementary material for: Purification of thonningianins A and B and four further derivatives from Thonningia sanguinea by one‐ and two‐dimensional centrifugal partition chromatography
Source: J Sep Sci. 2019 Nov 11;43(2):524–30. doi: 10.1002/jssc.201900811 (PMC7003852; doi:10.1002/jssc.201900811)
Supplement: Supplementary file 1 — Supporting Information [file JSSC-43-524-s001.docx]

**Supporting Information**

Purification of thonningianins A and B and four further derivatives from *Thonningia sanguinea* by one- and two-dimensional centrifugal partition chromatography

Luca Pompermaier^1^, Stefan Schwaiger^1, *^, Monizi Mawunu^2^, Thea Lautenschlaeger^3^, Hermann Stuppner^1^, Karine Faure^4^

^1^ Institute of Pharmacy/Pharmacognosy, Center for Molecular Biosciences Innsbruck, University of Innsbruck, 6020 Innsbruck, Austria

^2^ Kimpa Vita University, Province of Uíge, Uíge, Angola

^3^ Department of Biology, Institute of Botany, Faculty of Science, Technische Universität Dresden, 01217 Dresden, Germany

^4^ Université de Lyon, CNRS, Université Claude Bernard Lyon 1, Institut des Sciences Analytiques, F-69100 Villeurbanne, France

**Correspondence**

* Dr. Stefan Schwaiger, Research Associate, Institute of Pharmacy/Pharmacognosy, Center for Molecular Biosciences Innsbruck, University of Innsbruck, Innrain 80/82. E-mail: stefan.schwaiger@uibk.ac.at; Tel: +43 512 507 – 58409; Fax: +43 512 507 - 58499

Figure S1. UV chromatogram from Interchim software of the large scale (253 mL column) 1^st^ dimension CPC: **sample loading experiment with 780 mg extract.**


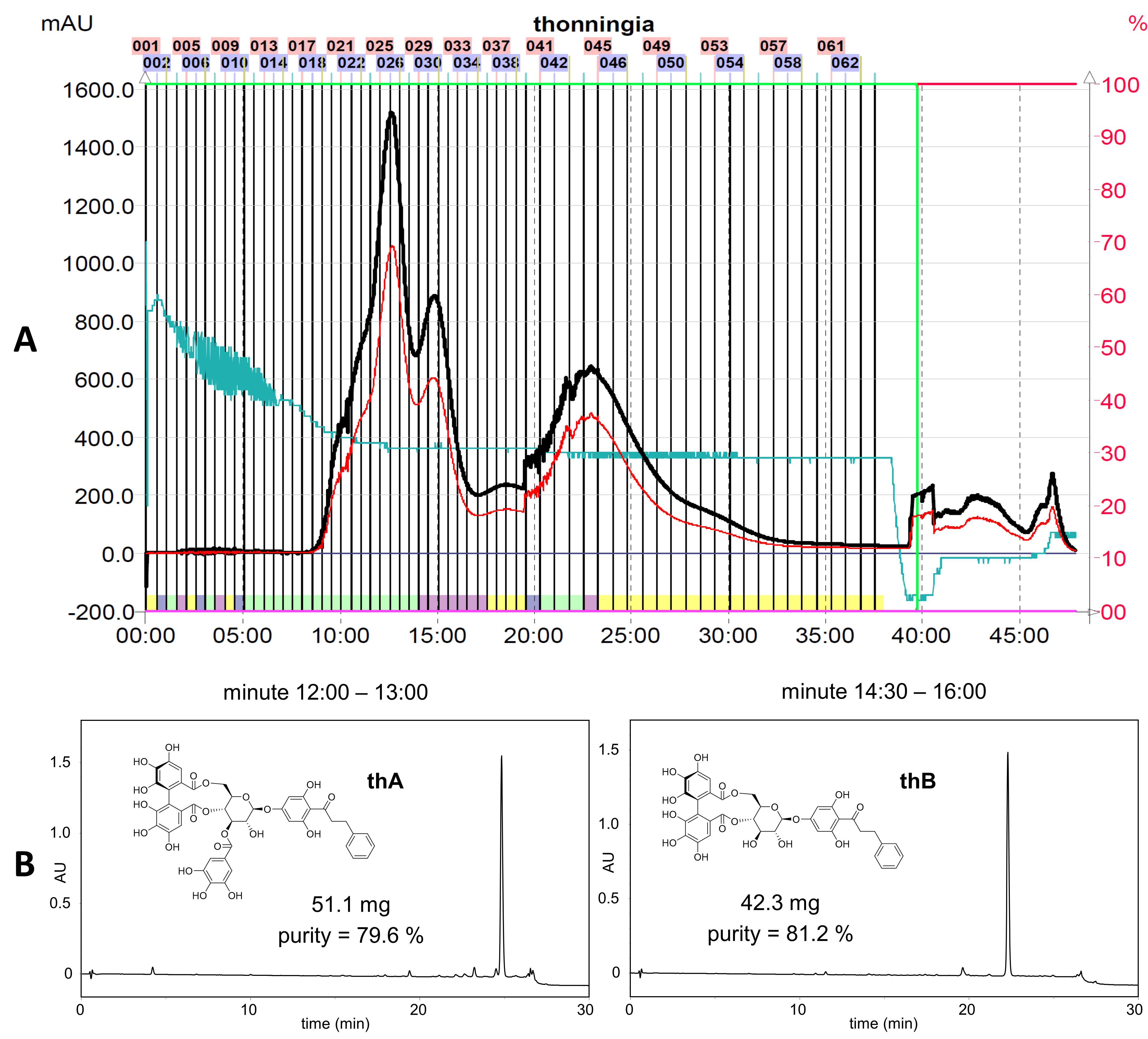


**A**: CPC-UV, parameters: solvent system = MTBE – DME – H_2_O (1:2:1); elution mode = ascending; flow rate = 20 mL/min; rotation speed = 2000 rpm; S_f_ = 66.8% prior to and 32.8% after injection; pressure at injection = 58 bar; sample = **780 mg** defatted MeOH extract in 18.9 mL upper/lower phase mixture; detection wavelengths = 220 nm (black line), 280 nm (red line); fraction collection = 10 mL (30 seconds) from minute 0:00 to 19:30, 15 mL (45 seconds) from minute 19:30 to 39:00; extrusion (without rotation) was started at minute 38:00.

**B**: HPLC-UV analysis (210 nm) of the fractions containing **thA** and **thB**. The tubes were pooled according to the indicated elution time frame.

Figure S2. Plot of partition coefficients for compounds **1**-**4** in the two selected solvent systems. 1^st^ dimension MTBE – DME – H_2_O (1:2:1) ascending mode; 2^nd^ dimension ethyl acetate – DME – H_2_O (2:1:1) descending mode.


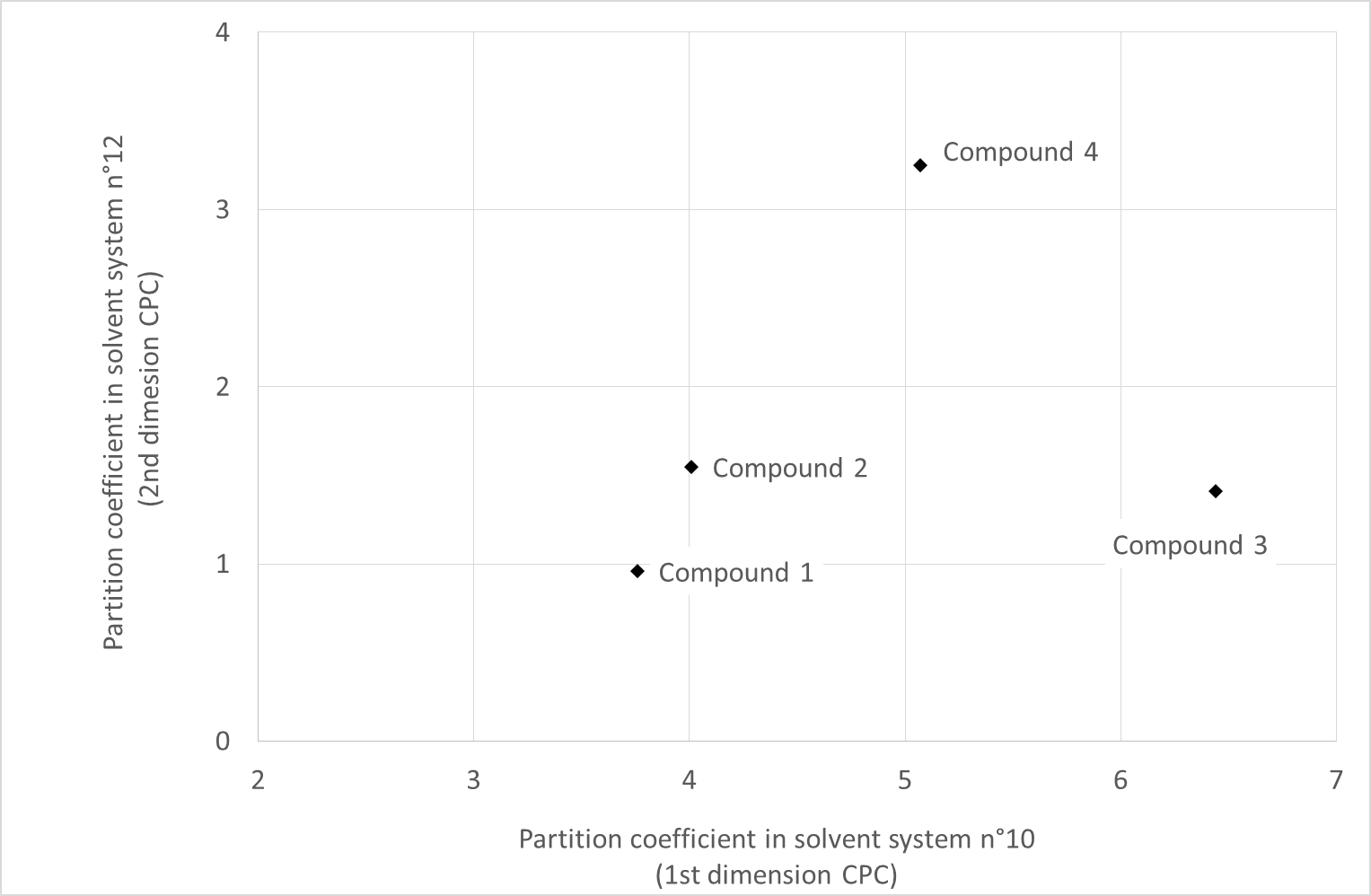


**Pretrial to increase the purity of thonningianin A and B by Sephadex LH20 column chromatography**

The performed CPC separation resulted in the isolation of 25.7 mg of **thA** (purity = 87.1%) and 21.1 mg of **thB** (purity = 85.0%). Since for some application the observed purity might be insufficient additional experiments were performed to increase the purity level. Purifications were performed by Sephadex LH20 CC (column length: 32 cm; Ø 1 cm, swollen in methanol for 24 h). The mobile phase was methanol. The eluate was collected in a time interval of 2 min corresponding to 1.5 mL. The separations were carried out with 20.0 mg **thA** (fraction I) and 15.0 mg **thB** (fraction II), respectively. The obtained fractions were analysed by TLC (mobile phase: EtOAc: MeOH: toluene: formic acid: water, 10:1:1:0.5:0.5, all v/v, see Figure S3 and S4) and combined to give three fractions per separation: **thonningianin A:** thA-S1 (tube 1-49) 3.20 mg; **thA-S2 (tube 50-71) 12.10 mg**; thA-S3 (tube 71-98) 2.70 mg; **thonningianin B:** thB-S1 (tube 1-29) 3.54 mg; **thB-S2 (tube 30-39) 8.80 mg**; thB-S3 (tube 40-65) 1.31 mg. Each obtained fraction was analysed by HPLC revealing a purity of 95.7% at 210 nm for fraction thA-S2 and 90.1% for fraction thB-S2 (see Figure S5 and S6).


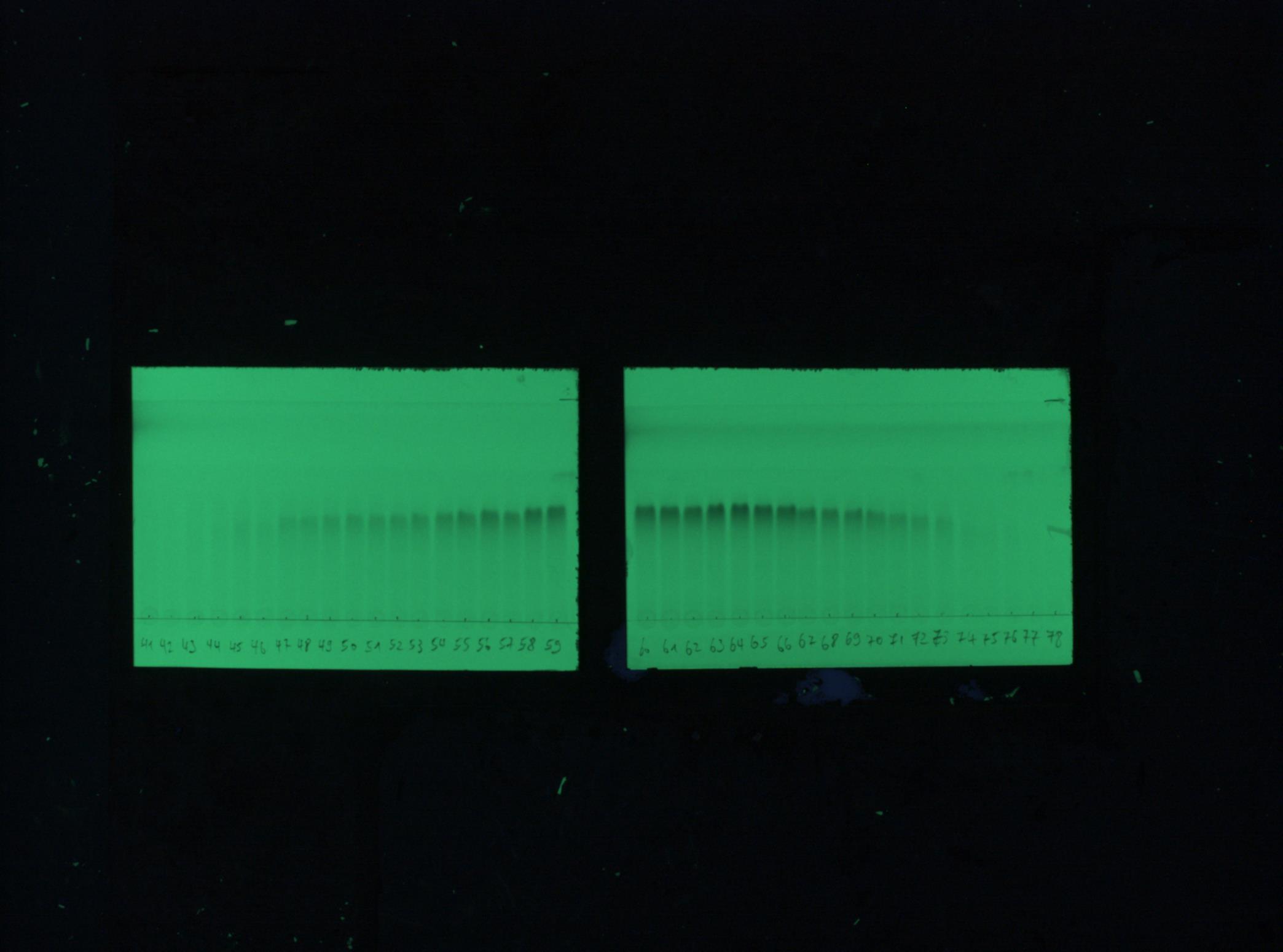

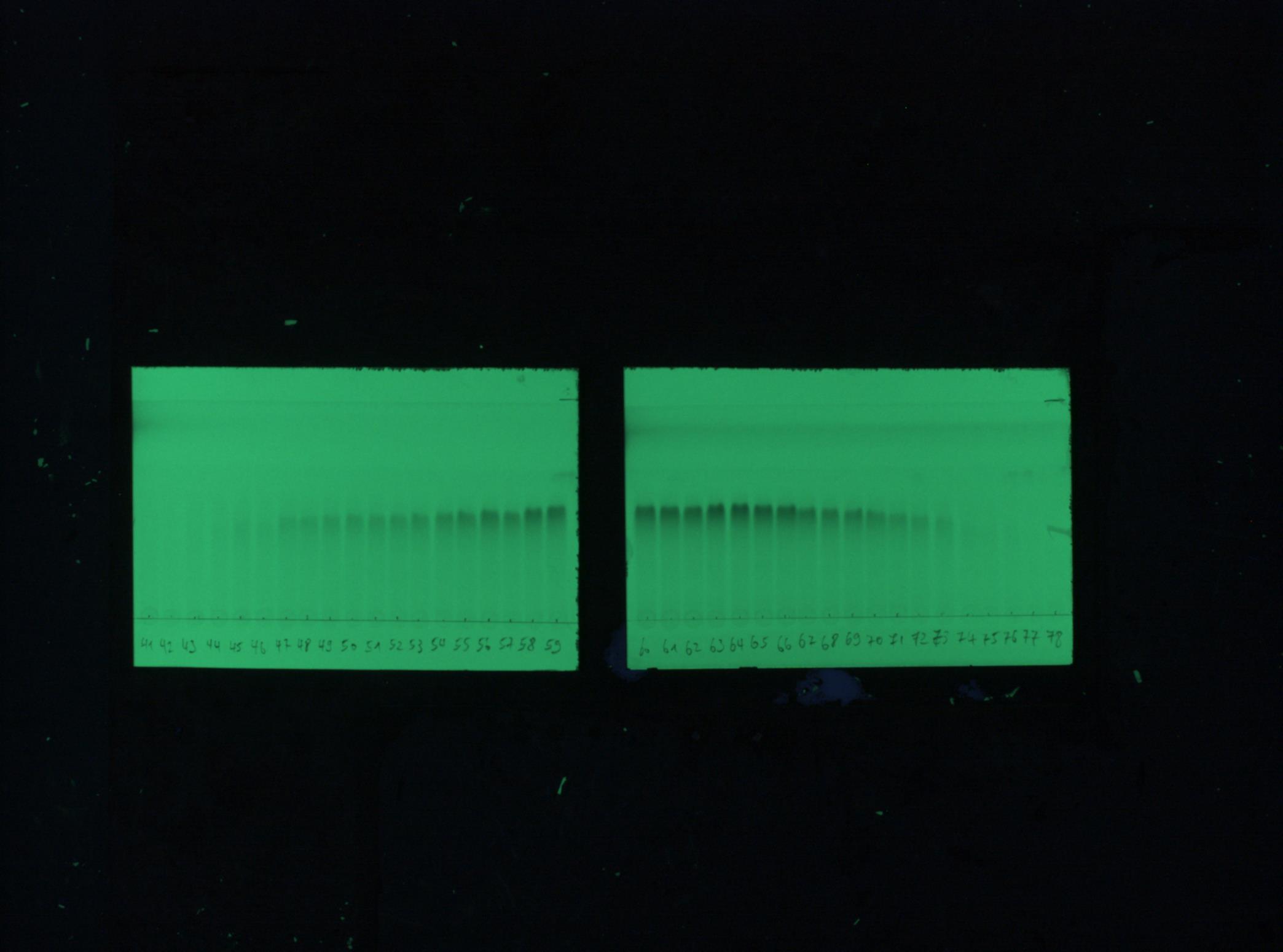


41 42 43 44 45 46 47 48 49 **50 51 52 53 54 55 56 57 58 59 60 61 62 63 64 65 66 67 68 69 70** 71 72 73 74 75 76 77 78

Figure S3. TLC analysis of the obtained Sephadex LH20 CC fractions of the separation of thonningianin A. TLC-parameter: applied sample volume: 10 µL per sample; mobile phase: EtOAc: MeOH: toluene: formic acid: water, 10:1:1:0.5:0.5, all v/v; stationary phase: TLC silica gel 60 F_254_ aluminum sheets (Merck) 6.7×10 cm; migration distance: 5.0 cm. TLCs were documented at 254 nm with a CAMAG Reprostar3 operated with winCATS software (2012).


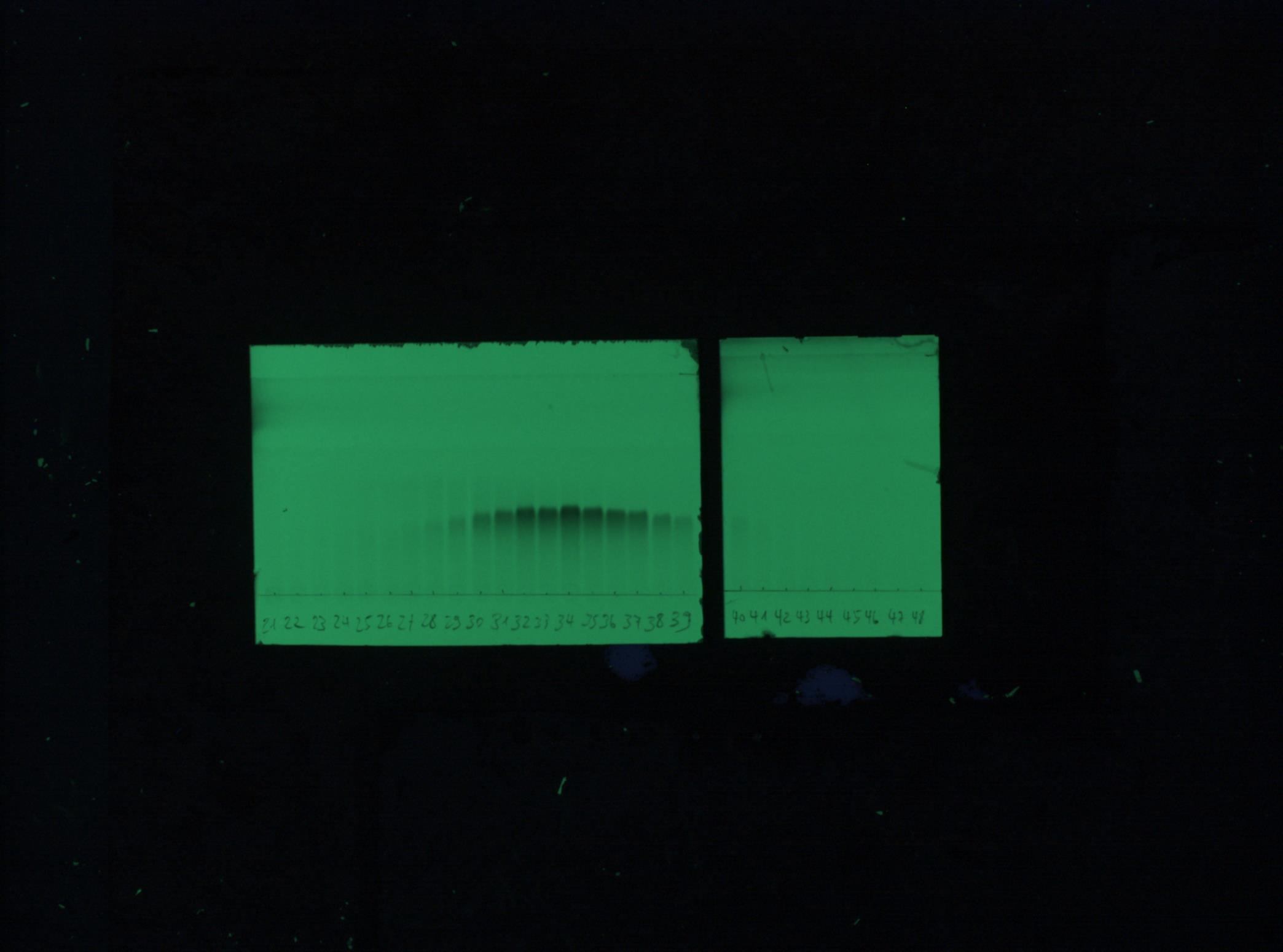

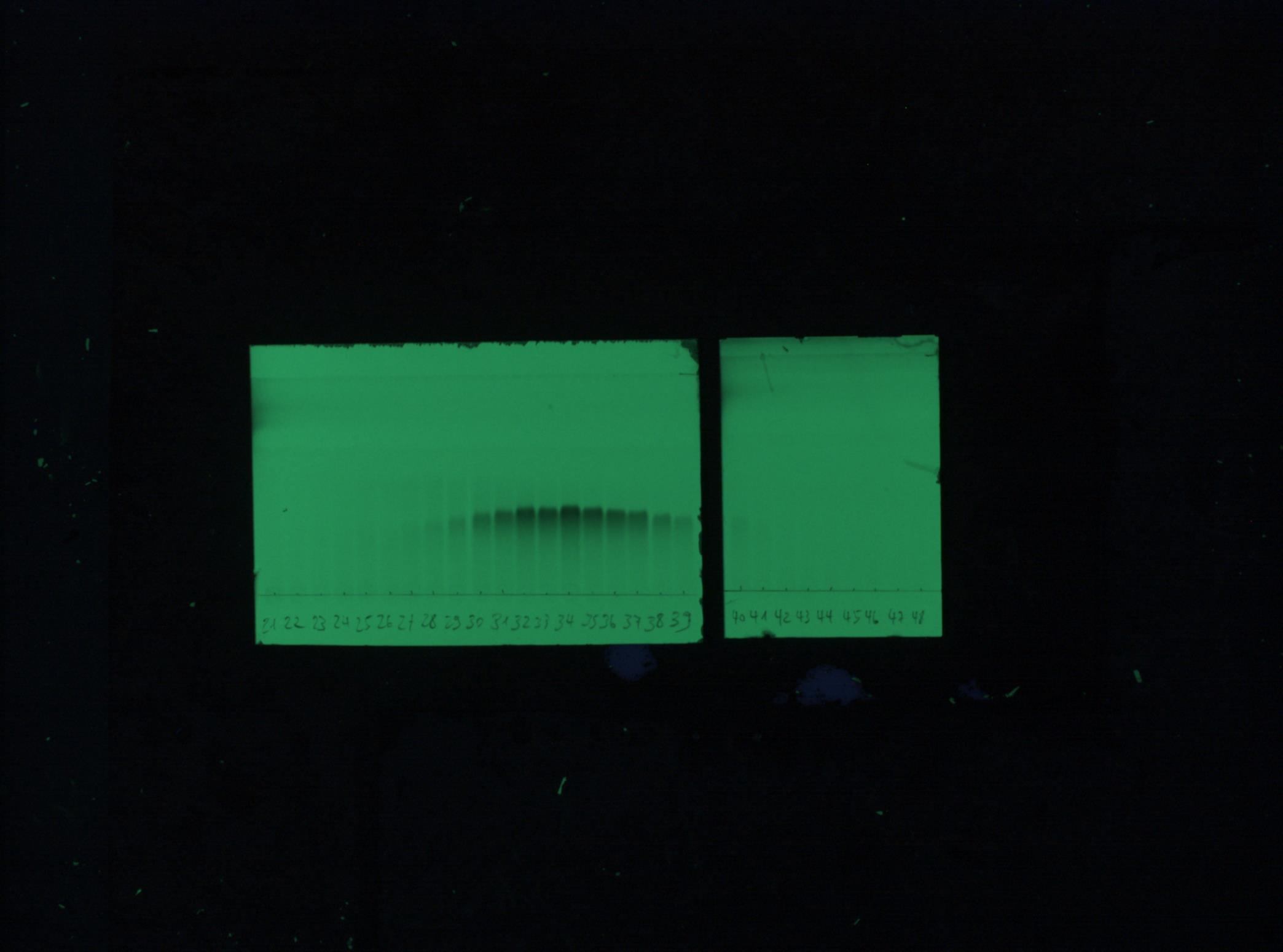


21 22 23 24 25 26 27 28 29 **30 31 32 33 34 35 36 37 38 39** 40 41 42 43 44 45 46 47 48

Figure S4. TLC analysis of the obtained Sephadex LH20 CC fractions of the separation of thonningianin B. TLC-parameter: applied sample volume: 10 µL per sample; mobile phase: EtOAc: MeOH: toluene: formic acid: water, 10:1:1:0.5:0.5, all v/v; stationary phase: TLC silica gel 60 F_254_ aluminum sheets (Merck) 6.7×10 cm; migration distance: 5.0 cm. TLCs were documented at 254 nm with a CAMAG Reprostar3 operated with winCATS software (2012).

**thA-S2**

λ = 210 nm

purity = 95.7%

min

5

10

15

20

25

mAU

0

200

400

600

800

1000

Figure S5. HPLC analysis of thA-S2 (0.5 mg/mL). HPLC: column: Agilent Eclipse XDB-C18 3.5 μm, 3.0 × 100 mm; mobile phase: A = H_2_O + 0.02% trifluoroacetic acid, B = acetonitrile; gradient: 0 min: 10% B, 25 min: 50% B, 26 min: 98% B, 30 min: stop (TS); temp: 30 °C; flow: 1 mL/min; inj. vol: 5 μL, blank run subtracted.

**thB-S2**

λ = 210 nm

purity = 90.1%

min

5

10

15

20

25

mAU

0

100

200

300

400

500

600

700

800

Figure S6. HPLC analysis of thB-S2 (0.5 mg/mL). HPLC: column: Agilent Eclipse XDB-C18 3.5 μm, 3.0 × 100 mm; mobile phase: A = H_2_O + 0.02% trifluoroacetic acid, B = acetonitrile; gradient: 0 min: 10% B, 25 min: 50% B, 26 min: 98% B, 30 min: stop (TS); temp: 30 °C; flow: 1 mL/min; inj. vol: 5 μL, blank run subtracted.
